# S49. Clinical activity and development of biomarkers for an engineered anti PDL1 antibody MPDL3280A

**DOI:** 10.1186/2051-1426-2-S2-I21

**Published:** 2014-03-12

**Authors:** E Cha

**Affiliations:** 1 Genentech, South San Francisco, CA 94080, USA

## Background

Human cancer cells may suppress the adaptive immune response by expressing PD-L1 and down-regulating T cell activity through PD-L1/PD-1 and PD-L1/B7.1 interactions. Disruption of PD-L1 signaling restores anti-tumor immunity, resulting in durable responses across multiple human tumor types. Here we describe the clinical activity and development of predictive biomarkers for MPDL3280A, a human monoclonal antibody with an engineered Fc-domain designed to optimize safety and efficacy, that targets PD-L1 and prevents binding to receptors PD-1 and B7.1.

## Materials & methods

We evaluated a multitude of biomarkers from pretreatment tumor specimens collected during clinical study of MPDL3280A. MPDL3280A has been administered as a monotherapy in over 300 pts with locally advanced or metastatic solid tumors in a phase 1 study.

## Results

To date, MPDL3280A has been well tolerated across multiple dose levels and no G3-5 pneumonitis has been reported. Responses were observed in multiple tumor types, including NSCLC, RCC, and melanoma, with ongoing responses seen in the majority of responders. We report the association between pretreatment immune-related markers and response to MPDL3280A.

## Conclusion

Overall, our assessment of tumor specimens not only has resulted in markers that may potentially identify response to MPDL3280A but also has furthered our understanding of the biologic activity of PD-L1 inhibition on treatment.

